# Predictors of self-reported research productivity amongst medical students in the United Kingdom: a national cross-sectional survey

**DOI:** 10.1186/s12909-023-04412-z

**Published:** 2023-06-06

**Authors:** Temidayo Osunronbi, William Adeboye, David Faluyi, Jasmine Sofela, Efua Abankwa, Semhar Abraha, Fatima Adamu-Biu, Zain Ahmad, Izieduwa Akhionbare, Chimba Chimba, Anna Chiara Corriero, Isata J. Fofanah, Ikenna Ibeanusi, Ummulkhulsum Ibrahim, Deborah Inyang, Robert Jones, Adebola Kolawole, Rachael Madume, Chenai Mandangu, Valentine Mberu, Ellen Nelson-Rowe, Marguerite O’Riordan, Serena Shoker, Agbolahan Sofela, REMED-U. K. collaborators, Dima Abdelhafiz, Ayanfe Adebayo, Oluwafemi Afolabi, Fatima Awow, Akua Crankson, Henry Exley, Lauren Frame, Ola Johnson, Risata A. Kufuor, William Madu, Calum McCutcheon, Christine Mitoko, Suaad Mohamed, Samantha R. Munyebvu, Max Shah, Oonagh Stewart, Alice Watts

**Affiliations:** 1 Melanin Medics Research Network, Luton, UK; 2 Department of Health Sciences, University of York, York, UK; 3 Faculty of Health, University of Plymouth, Plymouth, UK; 4 Southwest Neurosurgery Centre, University Hospitals Plymouth NHS Trust, Plymouth, PL6 8DH UK; 5 School of Medicine, University of Exeter Medical School, Exeter, EX1 2HZ UK

**Keywords:** Medical students, Research, United Kingdom, Productivity, Inequalities

## Abstract

**Background:**

The number of academic clinicians in the UK is declining and there are demographic inequalities in the clinical-academic workforce. Increased research productivity by medical students is believed to reduce future attrition in the clinical-academic workforce. Thus, this study investigated the association between student demographics and research productivity amongst UK medical students.

**Methods:**

This is a national multicentre cross-sectional study of UK medical students in the 2020/21 academic year. We appointed one student representative per medical school, and they disseminated a 42-item online questionnaire over nine weeks, through departmental emails and social media advertisements. The outcome measures were: (i) publications (yes/no) (ii) number of publications (iii) number of first-authored publications (iv) abstract presentation (yes/no). We utilised multiple logistic and zero-inflated Poisson regression analyses to test for associations between the outcome measures and predictor variables at a 5% significance level.

**Results:**

There are 41 medical schools in the UK. We received 1573 responses from 36 UK medical schools. We failed to recruit student representatives from three newly formed medical schools, whilst two medical schools prohibited us from sending the survey to their students. Women had lower odds of having a publication (OR: 0.53, 95% CI: 0.33–0.85) and on average had fewer first-author publications than men (IRR: 0.57, 95% CI: 0.37–0.89). Compared to white students, mixed-ethnicity students had greater odds of having a publication (OR: 3.06, 95% CI: 1.67–5.59), an abstract presentation (OR: 2.12, 95% CI: 1.37–3.26), and on average had a greater number of publications (IRR: 1.87, 95% CI: 1.02–3.43). On average, students who attended independent UK secondary schools had a higher rate of first-author publications compared to those that attended state secondary schools (IRR: 1.97, 95% CI: 1.23–3.15).

**Conclusion:**

Our data suggest that there are gender, ethnic and socioeconomic inequalities in research productivity among UK medical students. To tackle this, and potentially improve diversity in clinical academia, we recommend that medical schools should facilitate targeted high quality research mentorship, funding and training, especially for under-represented-in-medicine students.

**Supplementary Information:**

The online version contains supplementary material available at 10.1186/s12909-023-04412-z.

## Background

The phrase 'clinical academics' typically describes clinicians who are allotted protected time in their work schedules to engage in scholarly/research pursuits. The clinical academic role was created to facilitate the application and translation of research findings into clinical practice [1]. There has been a decline in the number of clinical academics in the United Kingdom (UK) [1]. In addition, the ethnic and gender profile of the clinical academic workforce is not reflective of the wider population of licensed doctors. Female clinicians and those from black, Asian and minority ethnic backgrounds have reduced access to research opportunities, with disproportionately low representation in the academic workforce and research leadership [1, 2].

Engaging medical students in research activities during their medical training could mitigate the declining number of academic clinicians [3]. Some authors argue that research experiences are incomplete unless they result in the dissemination of knowledge, usually through peer-reviewed publications and presentations at scientific meetings [4]. Moreover, medical students who publish their research are more likely to be scientifically active after graduation, thus reducing future attrition in the clinical academic workforce [5–7].

Differential attainment appears at medical school and persists after graduation [8]. Therefore, it is possible that the ethnic and gender imbalance seen in the clinical academic workforce is associated with the research experience of students in medical school. Tackling this inequality is important to ensure patients benefit from a diverse healthcare workforce [9, 10]. Hence, our study aims to investigate the associations between student demographics and research productivity among UK medical students. In this study, research productivity is defined as the product of research activities, namely publications and abstract presentations.

## Methods

This national multi-centre cross-sectional study received ethical approval from the Faculty of Health Research Ethics and Integrity Committee, University of Plymouth on 9 February 2021 (Ethics approval reference: 2570) and was conducted in line with the published protocol [11]. The participants were medical students aged 18 years or older who were enrolled in medical schools listed in the UK’s Medical Schools Council at the start of the 2020/21 academic year. All participants provided informed consent.

A 42-item questionnaire was created on Qualtrics™ based on literature findings [3, 12–22] and feedback from regional leads across 36 UK medical schools (Supplementary material 1). The questionnaire was distributed to the medical students using the UK National Research Collaborative Model, utilising regional leads to aid with survey dissemination Model [23]. We invited medical students from all UK medical schools to apply for regional leadership positions. The selected regional leads for each medical school were then tasked with sharing the online questionnaire with their respective medical student bodies through departmental e-mails and advertisements on student groups/forums across various social media platforms, with data collected over 9 weeks (22 March 2021 – 23 May 2021). At the time of this study, there were 41 medical schools in the UK. We were unable to recruit regional leads from three newly formed medical schools (University of Lincoln, University of Sunderland, and Edge Hill University). Two medical schools (University of Nottingham and University of Leicester) prohibited us from sending the survey to their students because they only allow surveys distributed directly from the UK Medical Schools Council.

We used the ‘RelevantID’ and ‘prevent multiple submissions’ features of Qualtrics™ to identify and prevent duplicate responses. The responses flagged as duplicates by Qualtrics™ were excluded from our analysis. Also, we excluded survey responses that were started but not completed by the respondents.

### Statistical analysis

Statistical analyses were conducted on Stata 17 software (StataCorp 2021). Frequencies were presented as both absolute numbers and percentages. Our outcome measures were the students' research output based on work(s) done since the beginning of their medical training: (i) PubMed-indexed publication (yes or no); (ii) Number of PubMed-indexed publications (excluding collaborator-status); (iii) Number of first-author PubMed-indexed publications; and (iv) abstract presentation at national/international conferences.

We investigated the following factors as predictors of the outcome measures: gender, ethnicity, number of research projects completed since starting medical school, perception of research, degree qualification prior to medical school, research experience prior to medical school, stage of medical training, part-time job, parental educational attainment, the type of secondary school attended (state school vs independent/private school vs secondary school outside the UK), and the type of university (Russell Group vs non-Russell Group). The Russell Group (Supplementary Material 1) is a self-selected association of 24 leading research-intensive universities in the United Kingdom, that are known for their high levels of research activity, strong teaching and learning environments, and commitment to innovation and engagement with industry and government. ‘Age’ was excluded as a predictor variable due to multicollinearity, while academic performance was excluded as a predictor variable because the response rate was less than 80%, as planned in our protocol [11].

We used cluster-robust standard errors in our analysis to account for student clusters within each university. Binary logistic regression was utilised to test for associations between the predictor variables and the following outcome measures: Publication (yes or no) and presentation (yes or no). Zero-inflated Poisson regression was utilised to test for associations between the predictor variables and the number of first-authored publications and the total number of publications. As planned in our protocol [11], we controlled for all the predictor variables in our multiple regression analyses. A *p*-value < 0.05 at 95% confidence interval was considered statistically significant.

## Results

### Demographics

At the time of this study, there was 42190 medical students in the UK [24]. We received 1797 responses, of which 154 responses were incomplete and excluded. Qualtrics™ identified 70 responses as duplicates, and these were excluded from our analysis. Of the 1573 valid responses collected, 66.0% (*n* = 1038) of the respondents were women, 32.5% (*n* = 512) were men, 1.1% (*n* = 17) identified as other, and 0.4% (*n* = 6) preferred not to indicate their gender (Table 1). This contrasts with the population of UK medical students, which comprises of 55% women and 45% men [24]. Most of our respondents were White (*n* = 798, 50.7%) or Asian (*n* = 411, 26.1%) (Table 1). Responses were obtained from thirty-six medical schools across the UK. Due to the inability to track the survey distribution, it was not possible to calculate a response rate. However, non-response bias was minimised by ensuring the questionnaire was disseminated through a range of platforms.

**Table 1:** Binary logistic regression (adjusted for clusters in universities) investigating the association between student characteristics and authorship on PubMed-indexed publications

**Variable**	**PubMed publication?**		**Crude estimates**		**Adjusted estimates**^a^	
	Yes (*n* = 145)	No (*n* = 1428)	OR (95% CI)	*P* value	OR (95% CI)	*P* value
**Gender**						
Woman (*n* = 1038)	76	962	0.53 (0.32; 0.89)	0.045*	0.53 (0.33; 0.85)	0.032*
Other (*n* = 17)	2	15	0.90 (0.20; 3.98)		0.51 (0.08; 3.29)	
Man (*n* = 512)	66	446	1 (Reference)		1 (Reference)	
**Ethnicity**						
Mixed (*n* = 105)	19	86	2.33 (1.43; 3.82)	< 0.001*	3.06 (1.67; 5.59)	< 0.001*
Asian (*n* = 411)	34	377	0.95 (0.63; 1.48)		1.04 (0.67; 1.60)	
Black (*n* = 206)	14	192	0.77 (0.40; 1.50)		0.93 (0.46; 1.89)	
Other (*n* = 48)	8	40	2.11 (0.76; 5.86)		3.10 (1.03; 9.28)	
White (*n* = 798)	69	729	1 (Reference)		1 (Reference)	
**Russell Group University**						
Yes (*n* = 889)	105	784	2.16 (1.16; 4.00)	0.015*	2.06 (0.99; 4.29)	0.054
No (*n* = 684)	40	644	1 (Reference)		1 (Reference)	
**Number of research projects done in medical school** (Median (Q1—Q3))	3 (2—5)	1 (0 – 2)	1.81 (1.50; 2.19)	< 0.001*	1.54 (1.30; 1.84)	< 0.001*
**Research perception** (Mean ± SD)	20.1 ± 4.0	18.4 ± 4.0	1.13 (1.05; 1.21)	0.001*	1.07 (1.00; 1.13)	0.038*
**Previous degree**						
Yes (*n* = 269)	32	237	1.42 (0.88; 2.29)	0.147	1.07 (0.56; 2.04)	0.838
No (*n* = 1304)	113	1191	1 (Reference)		1 (Reference)	
**Prior research experience**						
Yes (*n* = 675)	83	592	1.89 (1.22; 2.92)	0.004*	1.67 (1.06; 2.62)	0.028*
No (*n* = 898)	62	836	1 (Reference)		1 (Reference)	
**Stage of training**						
Clinical (*n* = 722)	122	600	7.32 (4.34; 12.35)	< 0.001*	4.78 (2.68; 8.52)	< 0.001*
Pre-clinical (*n* = 851)	23	828	1 (Reference)		1 (Reference)	
**Works/worked part-time**						
Yes (*n* = 830)	78	752	1.05 (0.71; 1.56)	0.815	0.72 (0.51; 1.03)	0.074
No (*n* = 711)	64	647	1 (Reference)		1 (Reference)	
**Parent has a degree**						
Yes (*n* = 1136)	110	1026	1.26 (0.85; 1.86)	0.250	0.86 (0.52; 1.43)	0.571
No (*n* = 395)	31	364	1 (Reference)		1 (Reference)	
**Secondary school**						
Independent (*n* = 283)	41	242	2.14 (1.33; 3.44)	0.007*	1.36 (0.87; 2.14)	0.356
Outside UK (*n* = 257)	28	229	1.54 (0.89; 2.68)		1.38 (0.69; 2.76)	
State (*n* = 1009)	74	935	1 (Reference)		1 (Reference)	

*OR* Odds ratio

^*^Significant at 5% significance level

^a^Adjusted for all the variables included in this table

### PubMed-indexed publications

**Fig. 1:**
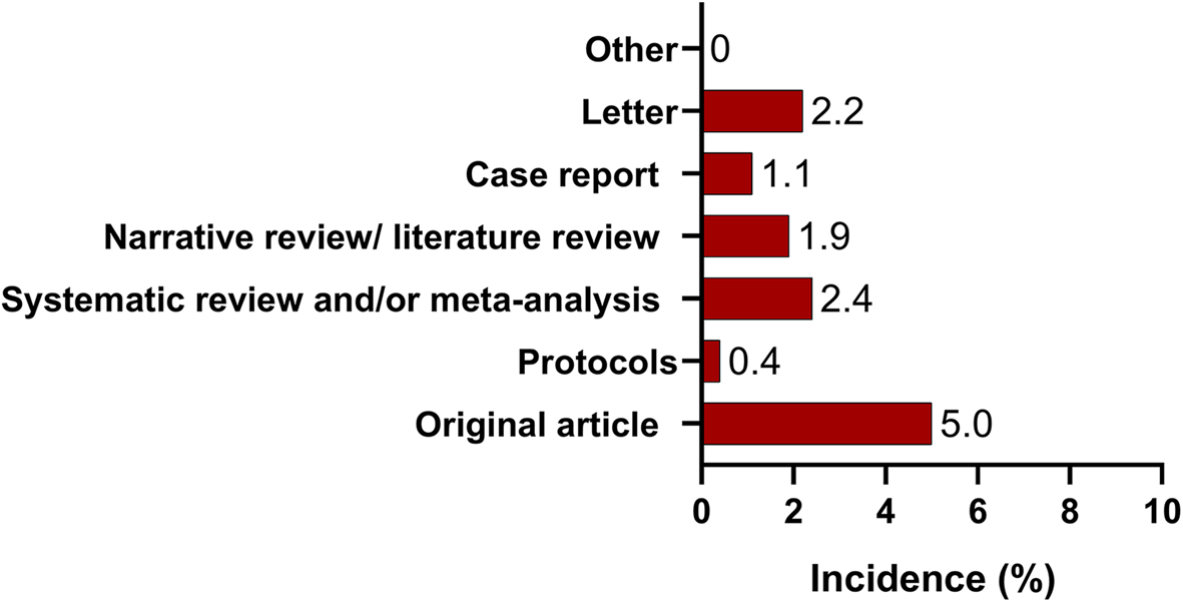
Type of publications authored by the respondents

One hundred and forty-five students (9.2%) had at least one PubMed-indexed publication. Original articles (5%) and systematic review/meta-analyses (2.4%) were the most common publications authored by the respondents (Fig. 1).

**Fig. 2:**
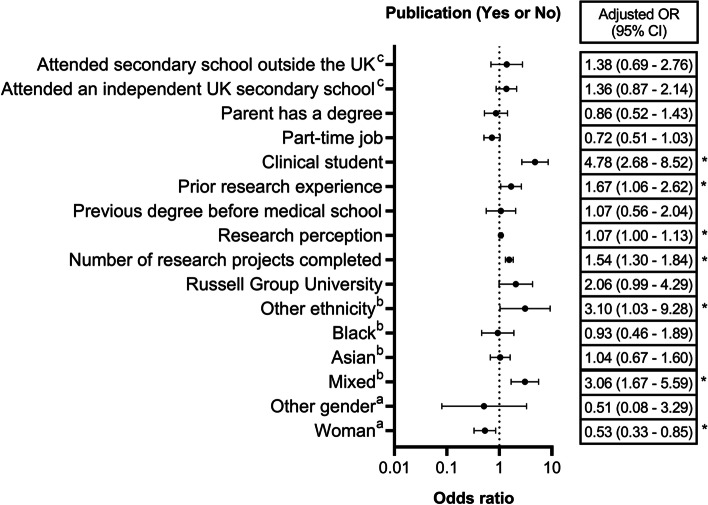
Adjusted odds ratio with 95% confidence interval for being an author on at least one PubMed-indexed article. References: ^a^man; ^b^white; ^c^state school. *: statistically significant

Multiple regression analysis (Table 1, Fig. 2) indicated that gender (*p* = 0.032), ethnicity (*p* < 0.001), the number of research projects conducted by the student (*p* < 0.001), perception of research (*p* = 0.038), prior research experience (*p* = 0.028), and stage of training (*p* < 0.001) independently influenced the odds of having at least one publication.

Compared to men, women had a 47% decrease in the odds of having at least one publication (adjusted OR: 0.53, 95% CI: 0.33 – 0.85). Compared to ‘white’ students, ‘mixed ethnicity’ students (adjusted OR: 3.06, 95% CI: 1.67 – 5.59) and ‘other ethnicity’ students (adjusted OR: 3.10, 95% CI: 1.03 – 9.28) had 3.06 times and 3.10 times greater odds of having at least one publication, respectively. There were no statistically significant differences between ‘white’ students and black/Asian ethnicities. Students in their clinical years (‘clinical-years students’) had 4.78 times greater odds of having a publication compared to ‘pre-clinical students’ (OR: 4.78, 95% CI: 2.68 – 8.52).

A higher number of completed research projects unit (adjusted OR: 1.54, 95% CI: 1.30 – 1.84) and a more positive belief in the value of research (adjusted OR: 1.07, 95% CI: 1.00 – 1.13) were associated with 54% and 7% increase, respectively, in the odds of having at least one publication. Students with prior research experience (before medical school) had 1.67 times greater odds of having a publication compared to those without prior experience (adjusted OR: 1.67, 95% CI: 1.06—2.62).

### Number of PubMed-indexed publications

**Fig. 3:**
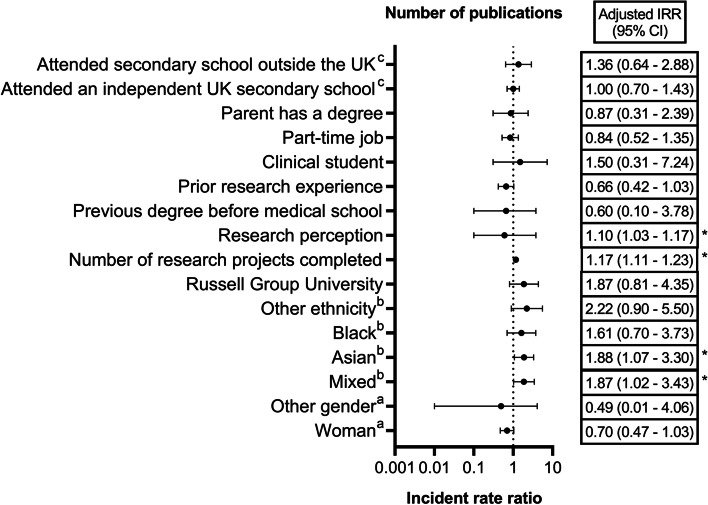
Adjusted incidence rate ratio with 95% confidence interval for the number of PubMed-indexed publications. References: ^a^man; ^b^white; ^c^state school. *: statistically significant

Multiple regression analysis (Table 2, Fig. 3) indicated that the number of research projects conducted by the student (*p* < 0.001) and the student’s perception of research (*p* = 0.003) independently influenced the number of PubMed-indexed publications authored by the student.

**Table 2:** Zero-inflated poisson regression (adjusted for clusters in universities) investigating the association between student characteristics and the number of PubMed-indexed publications

Variable	Crude estimates		Adjusted estimates^a^	
	IRR (95% CI)	*P* value	IRR (95% CI)	*P* value
**Gender**				
Woman (*n* = 1038)	0.54 (0.24; 0.88)	0.042*	0.70 (0.47; 1.03)	0.147
Other (*n* = 17)	0.39 (0.11; 1.40)		0.49 (0.01; 40.6)	
Man (*n* = 512)	1 (Reference)		1 (Reference)	
**Ethnicity**				
Mixed (*n* = 105)	1.00 (0.47; 2.15)	0.489	1.87 (1.02; 3.43)	0.073
Asian (*n* = 411)	1.59 (0.75; 3.38)		1.88 (1.07; 3.30)	
Black (*n* = 206)	0.69 (0.26; 1.82)		1.61 (0.70; 3.73)	
Other (*n* = 48)	1.16 (0.62; 2.18)		2.22 (0.90; 5.50)	
White (*n* = 798)	1 (Reference)		1 (Reference)	
**Russell Group University**				
Yes (*n* = 889)	1.51 (0.65; 3.51)	0.339	1.87 (0.81; 4.35)	0.145
No (*n* = 684)	1 (Reference)		1 (Reference)	
**Number of research projects done in medical school**	1.19 (1.16; 1.21)	< 0.001*	1.17 (1.11; 1.23)	< 0.001*
**Research perception**	1.02 (0.94; 1.11)	0.587	1.10 (1.03; 1.17)	0.003*
**Previous degree**				
Yes (*n* = 269)	0.50 (0.28; 0.90)	0.022*	0.60 (0.10; 3.78)	0.589
No (*n* = 1304)	1 (Reference)		1 (Reference)	
**Prior research experience**				
Yes (*n* = 675)	0.58 (0.38; 0.89)	0.012*	0.66 (0.42; 1.03)	0.065
No (*n* = 898)	1 (Reference)		1 (Reference)	
**Stage of training**				
Clinical (*n* = 722)	2.67 (1.08; 6.60)	0.033*	1.50 (0.31; 7.24)	0.614
Pre-clinical (*n* = 851)	1 (Reference)		1 (Reference)	
**Works/worked part-time**				
Yes (*n* = 830)	0.69 (0.36; 1.33)	0.267	0.84 (0.52; 1.35)	0.477
No (*n* = 711)	1 (Reference)		1 (Reference)	
**Parent has a degree**				
Yes (*n* = 1136)	1.33 (0.74; 2.37)	0.342	0.87 (0.31; 2.39)	0.784
No (*n* = 395)	1 (Reference)		1 (Reference)	
**Secondary school**				
Independent (*n* = 283)	0.87 (0.55; 1.38)	0.822	1.00 (0.70; 1.43)	0.713
Outside UK (*n* = 257)	0.99 (0.55; 1.81)		1.36 (0.64; 2.88)	
State (*n* = 1009)	1 (Reference)		1 (Reference)	

IRR: Incident rate ratio

^*^Significant at 5% significance level

^a^Adjusted for all the variables included in this table

For each additional research project conducted by a student, there were 1.17 times more PubMed-indexed articles published (adjusted IRR: 1.17, 95% CI: 1.11 – 1.23). A unit increase in the student’s perception of research was associated with 1.10 times increase in the rate of publication (adjusted IRR: 1.10, 95% CI: 1.03 – 1.17).

Overall, ethnicity was not a statistically significant predictor of the number of PubMed-indexed publications. However, we demonstrated that mixed-ethnicity students (adjusted IRR: 1.87, 95% CI: 1.02 – 3.43) and Asian students (adjusted IRR: 1.88, 95% CI: 1.07 – 3.30) had a rate 1.87 and 1.88 times greater than white students for the number of PubMed-indexed publications respectively.

### Number of first-author PubMed-indexed publications

**Fig. 4:**
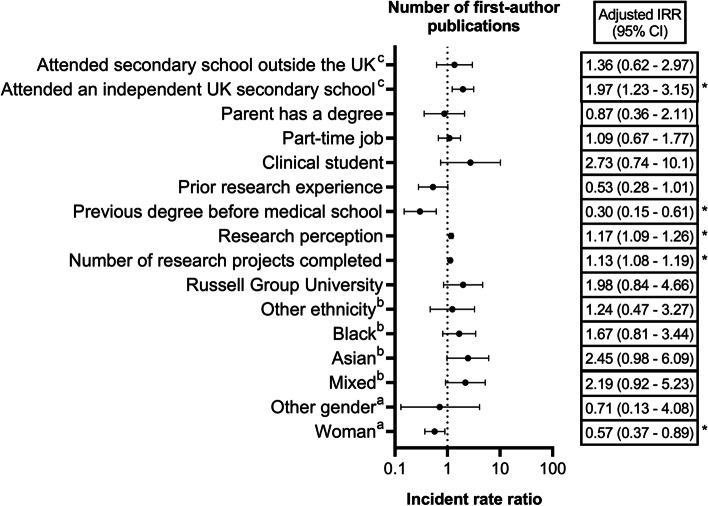
Adjusted incidence rate ratio with 95% confidence interval for the number of first-author PubMed-indexed publications. References: ^a^man; ^b^white; ^c^state school. *: statistically significant

Multiple regression analysis (Table 3, Fig. 4) indicated that gender (*p* = 0.020), the number of research projects conducted by the student (*p* < 0.001), the student’s perception of research (*p* < 0.001), completing a degree qualification before medical school (*p* = 0.001), and the type of secondary school attended (*p* = 0.018) independently influenced the number of first-author PubMed-indexed publications published by the student.

**Table 3:** Zero-inflated poisson regression (adjusted for clusters in universities) investigating the association between student characteristics and the number of first-author PubMed-indexed publications

**Variable**	**Crude estimates**		**Adjusted estimates**^a^	
	IRR (95% CI)	*P* value	IRR (95% CI)	*P* value
**Gender**				
Woman (*n* = 1038)	0.54 (0.26; 1.11)	0.002*	0.57 (0.37; 0.89)	0.020*
Other (*n* = 17)	0.09 (0.03; 0.35)		0.71 (0.13; 4.08)	
Man (*n* = 512)	1 (Reference)		1 (Reference)	
**Ethnicity**				
Mixed (*n* = 105)	1.16 (0.32; 4.13)	0.520	2.19 (0.92; 5.23)	0.272
Asian (*n* = 411)	1.65 (0.54; 5.02)		2.45 (0.98; 6.09)	
Black (*n* = 206)	1.58 (0.32; 7.82)		1.67 (0.81; 3.44)	
Other (*n* = 48)	0.70 (0.12; 3.98)		1.24 (0.47; 3.27)	
White (*n* = 798)	1 (Reference)		1 (Reference)	
**Russell Group University**				
Yes (*n* = 889)	1.45 (0.35; 5.97)	0.603	1.98 (0.84; 4.66)	0.118
No (*n* = 684)	1 (Reference)		1 (Reference)	
**Number of research projects done in medical school**	1.15 (1.09; 1.21)	< 0.001*	1.13 (1.08; 1.19)	< 0.001*
**Research perception**	1.09 (1.00; 1.20)	0.051	1.17 (1.09; 1.26)	< 0.001*
**Previous degree**				
Yes (*n* = 269)	0.17 (0.04; 0.71)	0.016*	0.30 (0.15; 0.61)	0.001*
No (*n* = 1304)	1 (Reference)		1 (Reference)	
**Prior research experience**				
Yes (*n* = 675)	0.38 (0.19; 0.77)	0.008*	0.53 (0.28; 1.01)	0.055
No (*n* = 898)	1 (Reference)		1 (Reference)	
**Stage of training**				
Clinical (*n* = 722)	7.47 (1.04; 53.46)	0.045*	2.73 (0.74; 10.12)	0.133
Pre-clinical (*n* = 851)	1 (Reference)		1 (Reference)	
**Works/worked part-time**				
Yes (*n* = 830)	0.80 (0.31; 2.11)	0.659	1.09 (0.67; 1.77)	0.737
No (*n* = 711)	1 (Reference)		1 (Reference)	
**Parent has a degree**				
Yes (*n* = 1136)	1.39 (0.54; 3.57)	0.489	0.87 (0.36; 2.11)	0.757
No (*n* = 395)	1 (Reference)		1 (Reference)	
**Secondary school**				
Independent (*n* = 283)	0.91 (0.52; 1.59)	0.946	1.97 (1.23; 3.15)	0.018*
Outside UK (*n* = 257)	0.96 (0.23; 4.00)		1.36 (0.62; 2.97)	
State (*n* = 1009)	1 (Reference)		1 (Reference)	

*IRR* Incident rate ratio

^*^Significant at 5% significance level

^a^Adjusted for all the variables included in this table

The rate of first-author publications among women was 0.57 times lower compared to men (adjusted IRR: 0.57, 95% CI: 0.37 – 0.89). Compared to the students that attended UK state secondary schools, those that attended independent UK secondary schools had a rate 1.97 times greater for first-author publications (adjusted IRR: 1.97, 95% CI: 1.23—3.15). Compared to other students, those that completed a degree qualification before medical school had a rate 0.30 times lower for first-author publications (adjusted IRR: 0.30, 95% CI: 0.15—0.61).

For every extra research project conducted, students published 1.13 times more first-author PubMed-indexed articles (adjusted IRR: 1.13, 95% CI: 1.08 – 1.19). A unit increase in the student’s research perception was associated with 1.17 times increase in the rate of first-author publications (adjusted IRR: 1.17, 95% CI: 1.09 – 1.26).

### National/international presentations

Three hundred and forty-two students (21.7%) had at least one oral/poster presentation at national/international level.

**Fig. 5:**
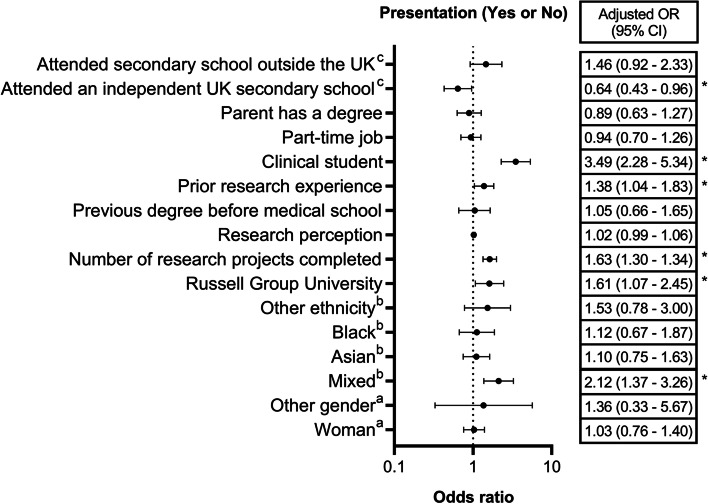
Adjusted odds ratio with 95% confidence interval for having at least one national/ international presentation. References: ^a^man; ^b^white; ^c^state school. *: statistically significant

Multiple regression analysis (Table 4, Fig. 5) indicated that ethnicity (*p* = 0.019), university type (*p* = 0.024), the number of research projects conducted by the student (*p* < 0.001), prior research experience (*p* = 0.025), stage of training, and the type of secondary school attended (*p* = 0.029) independently influenced the odds of having at least one presentation at national/international level.

**Table 4:** Binary logistic regression (adjusted for clusters in universities) investigating the association between student characteristics and national/international abstract presentation

Variable	Oral/poster presentation?		Crude estimates		Adjusted estimates^a^	
	Yes	No	OR (95% CI)	*P* value	OR (95% CI)	*P* value
**Gender**						
Woman (*n* = 1038)	220	818	0.92 (0.69; 1.23)	0.450	1.03 (0.76; 1.40)	0.888
Other (*n* = 17)	6	11	1.86 (0.62; 5.56)		1.36 (0.33; 5.67)	
Man (*n* = 512)	116	396	1 (Reference)		1 (Reference)	
**Ethnicity**						
Mixed (*n* = 105)	34	71	1.77 (1.14; 2.75)	0.096	2.12 (1.37; 3.26)	0.019*
Asian (*n* = 411)	84	327	0.95 (0.67; 1.33)		1.10 (0.75; 1.63)	
Black (*n* = 206)	43	163	0.97 (0.65; 1.47)		1.12 (0.67; 1.87)	
Other (*n* = 48)	11	37	1.10 (0.56; 2.17)		1.53 (0.78; 3.00)	
White (*n* = 798)	170	628	1 (Reference)		1 (Reference)	
**Russell Group University**						
Yes (*n* = 889)	236	653	1.95 (1.34; 2.83)	< 0.001*	1.61 (1.07; 2.45)	0.024
No (*n* = 684)	107	577	1 (Reference)		1 (Reference)	
**Number of research projects done in medical school**			1.88 (1.54; 2.31)	< 0.001*	1.63 (1.34; 1.99)	< 0.001*
**Research perception**			1.07 (1.03; 1.11)	< 0.001*	1.02 (0.99; 1.06)	0.242
**Previous degree**						
Yes (*n* = 269)	68	201	1.27 (0.84; 1.91)	0.258	1.05 (0.66; 1.65)	0.848
No (*n* = 1304)	275	1029	1 (Reference)		1 (Reference)	
**Prior research experience**						
Yes (*n* = 675)	175	500	1.52 (1.21; 1.91)	< 0.001*	1.38 (1.04; 1.83)	0.025*
No (*n* = 898)	168	730	1 (Reference)		1 (Reference)	
**Stage of training**						
Clinical (*n* = 722)	259	463	5.11 (3.20; 8.13)	< 0.001*	3.49 (2.28; 5.34)	< 0.001*
Pre-clinical (*n* = 851)	84	767	1 (Reference)		1 (Reference)	
**Works/worked part-time**						
Yes (*n* = 830)	201	629	1.35 (1.06; 1.72)	0.015*	0.94 (0.70; 1.26)	0.684
No (*n* = 711)	136	575	1 (Reference)		1 (Reference)	
**Parent has a degree**						
Yes (*n* = 1136)	257	879	1.15 (0.82; 1.61)	0.409	0.89 (0.63; 1.27)	0.528
No (*n* = 395)	80	315	1 (Reference)		1 (Reference)	
**Secondary school**						
Independent (*n* = 283)	63	220	1.12 (0.81; 1.55)	0.159	0.64 (0.43; 0.96)	0.029*
Outside UK (*n* = 257)	72	185	1.53 (0.98; 2.39)		1.46 (0.92; 2.33)	
State (*n* = 1009)	205	804	1 (Reference)		1 (Reference)	

*OR* Odds ratio

^*^Significant at 5% significance level

^a^Adjusted for all the variables included in this table

Compared to ‘white’ students, ‘mixed ethnicity’ students had 2.12 (adjusted OR: 2.12, 95% CI: 1.37 – 3.26) times greater odds of having at least one presentation. There were no statistically significant differences between ‘white’ students and black/Asian/other ethnicity students. Those that attended independent UK secondary schools compared to state UK secondary schools had a 36% decrease in their odds of having at least one presentation (adjusted OR: 0.64, 95% CI: 0.43 – 0.96).

Clinical students had 3.49 times greater odds of having at least one presentation compared to preclinical students (adjusted OR: 3.49, 95% CI: 2.28 – 5.34). Those with prior research experience (adjusted OR: 1.38, 95% CI: 1.04 – 1.83) and the students at Russell Group universities (adjusted OR: 1.61, 95% CI: 1.07 – 2.45) had 1.38 times and 1.61 times greater odds of having a presentation compared to their counterparts, respectively. A unit increase in the number of research projects completed was associated with a 63% increase in the odds of having at least one presentation (adjusted OR: 1.63, 95% CI: 1.34 – 1.99).

## Discussion

In this study, we investigated the self-reported factors influencing research productivity (publications and presentations) amongst students across 36 UK medical schools. We found that gender, ethnicity, type of secondary school attended, number of research projects completed, perception of research, research experience before medical school, and stage of medical training influenced the publication and presentation practices amongst medical students.

In corroboration with the literature [3], our study demonstrates that compared to men, women were less likely to have a publication and on average, had fewer first-author publications. This is consistent with the generalised gender gap in the authorship of academic medical articles [25]. The quality of research mentorship received by a student has a substantial influence on their research productivity [26]. Previous studies have reported that women are less likely to work with successful research mentors [26, 27] and that women may receive inferior mentoring [28, 29], but it is unknown if this is because women are less likely to approach successful research mentors or are more likely to be rejected by these mentors [26]. Hence, facilitating the selection of good quality research mentors for women may improve their research productivity in medical school [30].

We found that compared to white students, those that identified as mixed ethnicity were more likely to have a PubMed-indexed publication and abstract presentation as well as a greater number of publications. Homophily is well recognised in medical education [31, 32], and it has been reported that people of similar ethnicity are more likely to co-author scientific papers together than with people of other ethnicities [33]. Some authors report that although most people who are of mixed ethnicity identify as being bi- or multi-racial, some may identify themselves as mono-racial in some contexts [34, 35]. Possibly, the racial fluidity of mixed-race students enables them to form social networks with a diverse group of peers and research mentors [36]. Some authors have reported an association between medical students’ social networks and their academic performance [31], and this is a possible explanation for the greater level of research productivity amongst mixed race students. Encouraging underrepresented minority students to partake in funded summer studentships is reported to improve publication rates in this group [37], and this could mitigate the ethnic gap in the research productivity found in our study.

Compared to preclinical students, the students in their clinical years of training had greater odds of having a publication and an abstract presentation. This is similar to findings in the literature, and this could be attributed to greater time allowance, more attainment of research-specific skills and more exposure to research mentors over the years [4, 16, 38, 39]. In addition, we found that attending a Russell Group (research-intense) university increases the odds of having an abstract presentation but does not influence the publication metrics. Previous studies reported that ‘research-elite’ universities offer more opportunities for student research [40] with these students seemingly having a more satisfactory research training experience [17].

On average, the students that attended an independent/private UK secondary school had approximately twice the number of first-author publications of those that attended UK state schools. This finding mirrors previous reports that the odds of entering a UK medical school are doubled by attending an independent UK secondary school rather than a state school [41]. This advantage may in part be because independent schools are more likely to provide better academic support and offer activities that improve the students’ research skills before starting medical training. We observed no difference in co-authorship by the type of secondary school attended, but interestingly, those that attended independent secondary schools had lesser odds of presenting an abstract. Compared to being a co-author or presenting an abstract, being a first author on a publication requires a higher level of project responsibility, contribution and research skills. Research mentors/supervisors may offer these first-author projects to those who have been pre-trained in research skills and knowledge. This idea is supported by our finding that those that had research experience before medical school had greater odds of being published authors and presenting abstracts. Hence, the gap in research productivity between the students that attended state and independent secondary schools could be narrowed by organising summer studentships that provide academic/ research enrichment in medical sciences for state secondary school students [42].

### Limitations

To our knowledge, this is the largest study investigating the factors that influence research productivity amongst UK medical students. The questionnaire approach increased the scope of our study but limited its depth as we were unable to verify how the identified factors influence research productivity. Hence, future studies could utilise a qualitative focus group/interview approach.

Another limitation of our study is that student-authored manuscripts under review (submitted) and submitted presentation abstracts were not included, and this may have affected the analysis of research productivity amongst the students. As this questionnaire was not compulsory for students to complete, we recognise that there may be a degree of selection bias. Research-oriented students are more likely to respond to survey, hence, this survey may not be a true representation of medical students in the UK. Similar to previous surveys of UK medical students [16, 43, 44], 66% of participants were women, in comparison to 55% of UK medical students who are women. Thus, the results may not be generalisable to the UK medical student population. Lastly, this is a self-reporting study which is liable to recall bias, and the anonymity of the respondents meant it was not possible to verify their responses independently.

## Conclusion

The gender and ethnic disparities in the academic-clinician workforce is reflected in the current UK medical student population. Women are less likely to publish their research work and on average have fewer first-author publications compared to men. We found that the students that identified as mixed ethnicity had higher research productivity across all metrics than other students, and showed that on average, the students that attended an independent UK secondary school had more first-author publications than those that attended state secondary schools.

To reduce the disparities in research productivity amongst medical students, we recommend that medical schools: (i) facilitate the selection of good quality research mentors for medical students; (ii) encourage underrepresented medical students to partake in funded summer studentships; and (iii) organise academic/research enrichment programmes for state secondary school students.

## Supplementary Information

**Additional file 1. **

## Data Availability

The datasets generated and/or analysed during the current study are not publicly available due to ethical concerns and the conditions for this study’s registration at our institution but are available from the corresponding author on reasonable request.

## Abbreviations

CI: Confidence interval
GDPR: General Data Protection Regulation
IRR: Incident rate ratio
OR: Odds ratio
UK: United Kingdom
