# Supplementary material for: Predictors of self-reported research productivity amongst medical students in the United Kingdom: a national cross-sectional survey
Source: BMC Med Educ. 2023 Jun 6;23:412. doi: 10.1186/s12909-023-04412-z (PMC10243073; doi:10.1186/s12909-023-04412-z)
Supplement: Supplementary file 1 — Additional file 1. [file 12909_2023_4412_MOESM1_ESM.pdf]

QUESTIONNAIRE

Start of Block: Introduction

Q1

Factors influencing **R**esearch **E**ngagement among **Med**ical students in the **U**nited **K**ingdom

Survey Lead: Melanin Medics

Survey Criteria:
Participants must be 18 years or older and should be enrolled in a UK medical school by the start of the 2020/21 academic year

Aims: This study aims to investigate the factors affecting UK medical students' involvement in research, and to understand their perceptions of research, motivation for and the barriers to their engagement in research. We also aim to produce evidence-based reports that will guide UK medical schools and relevant organisations in developing resources and programmes that will increase the number of future academic clinicians and resolve inequalities in research opportunities and engagement amongst future clinicians.
Ethics: This study received ethical approval from the Faculty of Health Research Ethics and Integrity Committee, University of Plymouth on the 9th February 2021 (Ethics approval reference: 2570).
Consent, confidentiality, and anonymity:
All responses recorded will be anonymous and will be held in confidence according to the General Data Protection Regulation (GDPR) guidelines and there are 'prefer not to answer' options for questions that may be deemed sensitive.
Data will be held for a period of ten years from the publication of the research results. Due to the anonymity of data collected in this study, it is technically impossible to withdraw your answers following submission. Hence, submitting the questionnaire is taken as providing informed consent. However, you can withdraw your consent before submission, at any point whilst completing the questionnaire, and your responses will be excluded from our analysis.

Any questions, concerns, complaints, or requests regarding the study can be sent to academia@melaninmedics.org or Dr Temidayo Osunronbi (temidayo@melaninmedics.org).

It would take you approximately **10 to 15 minutes** to complete this survey.

Q2 **I <u>consent</u> to participate in this survey and for my anonymised data to be used for future research.**

○ Yes

○ No (this will terminate the survey)

End of Block: Introduction

Start of Block: Demographics

*

Q3 **Age (in years)**

____

Q4 **Gender**

○ Man

○ Woman

○ Non-binary

○ Other (please specify in the box below)
____

○ Prefer not to answer

Q5 **What is your ethnic group?**

- ○ White
- ○ Mixed/ Multiple ethnic groups
- ○ Asian/ Asian British
- ○ Black/ African / Caribbean/ Black British
- ○ Other ethnic group (please specify in the box below)

____

Q6 **University of Study**

- ◯ University of Aberdeen School of Medicine and Dentistry
- ◯ Anglia Ruskin University School of Medicine
- ◯ Aston University Medical School
- ◯ Barts and The London School of Medicine and Dentistry
- ◯ University of Birmingham College of Medical and Dental Sciences
- ◯ Brighton and Sussex Medical School
- ◯ University of Bristol Medical School
- ◯ University of Buckingham Medical School
- ◯ University of Cambridge School of Clinical Medicine
- ◯ Cardiff University School of Medicine
- ◯ University of Dundee School of Medicine
- ◯ Edge Hill University Medical School
- ◯ The University of Edinburgh Medical School
- ◯ University of Exeter Medical School
- ◯ University of Glasgow School of Medicine
- ◯ Hull York Medical School
- ◯ Imperial College London Faculty of Medicine
- ◯ Keele University School of Medicine
- ◯ Kent and Medway Medical School
- ◯ King's College London GKT School of Medical Education

- Lancaster University Medical School
- University of Leeds School of Medicine
- University of Leicester Medical School
- University of Liverpool School of Medicine
- University of Manchester Medical School
- Newcastle University School of Medical Education
- Norwich Medical School
- University of Nottingham School of Medicine
- University of Nottingham - Lincoln Medical School
- University of Oxford Medical Sciences Division
- Plymouth University Peninsula Schools of Medicine and Dentistry
- Queen's University Belfast School of Medicine
- University of Sheffield Medical School
- University of Southampton School of Medicine
- University of St Andrews School of Medicine
- St George's, University of London
- University of Sunderland School of Medicine
- Swansea University Medical School
- University of Central Lancashire School of Medicine
- University College London Medical School
- University of Warwick Medical School

Q7 **Did you complete a degree before starting medical school?**

- ◯ Yes
- ◯ No

Display This Question:

If Did you complete a degree before starting medical school? = Yes

Q8 **What was your highest level of qualification before starting medical school?**

- ◯ BSc or equivalent
- ◯ BDS
- ◯ MSc/ MPhil or equivalent
- ◯ PhD/ DPhil or equivalent
- ◯ Other ____

Q9 **What type of medical course are you on?**

- ◯ 4-year graduate entry programme
- ◯ 5-year programme
- ◯ 6-year programme (excluding medicine with foundation year and extended programmes)
- ◯ 6-year medicine with foundation year/ extended programme
- ◯ Medicine Maxfax Entry Programme

Q10 **What year of medical school (<u>excluding intercalation year</u>) are you in?**

- ◯ Foundation Year (of medical school)
- ◯ Year 1
- ◯ Year 2
- ◯ Year 3
- ◯ Year 4
- ◯ Year 5

Q11 **Have you intercalated or currently intercalating?**

- ◯ Yes
- ◯ No

Q12 **What is your current medical school performance decile score? (Ranging from 1st decile (top 10%) to 10th decile (bottom 10%))**

- ◯ 1st
- ◯ 2nd
- ◯ 3rd
- ◯ 4th
- ◯ 5th
- ◯ 6th
- ◯ 7th
- ◯ 8th
- ◯ 9th
- ◯ 10th
- ◯ Unknown
- ◯ Prefer not to answer

Q13
What type of school did you mainly attend between the ages of 11 and 16?

○ State school in the UK

○ Independent school in the UK

○ Attended school outside the UK

○ Other (please specify in the box below)
____

○ Not sure

○ Prefer not to say

Q14 **Since starting medical school, have you worked part-time at any point to fund your studies and/or maintenance costs?**

○ Yes

○ No

○ Prefer not to say

Q15
Has any of your parent(s) or guardian(s) completed a university degree course or equivalent (eg, BA, BSc or higher)?

○ Yes

○ No

○ Not sure

○ Prefer not to say

End of Block: Demographics

Start of Block: Research engagement

Q16 This study defines research engagement as contributing to <u>at least one</u> of the following actions in a project - Formulating a research question - Conducting a structured literature search - Designing methodology - Ethical approval application - Data collection (quantitative or qualitative data) - Data analysis (qualitative or quantitative data) - Presenting result (oral or poster) - Writing a report/manuscript

---

*

Q17 **Using the aforementioned definition of research engagement, what research experience did you have <u>before entering</u> medical school?** (Please tick all that apply)

- ▢ Lab/field research
- ▢ Extended Project Qualification (EPQ)
- ▢ Research assistant
- ▢ Other (please specify in the box below)

____

- ▢ None

---

*

Q18
Using the aforementioned definition of research engagement, <u>since starting</u> medical school, what type of research projects have you contributed to? (Please tick all that apply) Note that the

research work could be through your medical school (e.g. student selected components/modules) and/or other means. The project may or may not have been presented or published.

- ☐ Qualitative research
- ☐ Laboratory-based
- ☐ Case report
- ☐ Observational study
- ☐ Randomised controlled trials (RCT)
- ☐ Other interventional studies (excluding RCT)
- ☐ Systematic review/ meta-analysis
- ☐ Narrative/ literature review
- ☐ Others (excluding audits) (please specify in the box below)
____
- ☐ None

Skip To: End of Block If Using the aforementioned definition of research engagement, since starting medical school, what... = None

Q19 **Since starting medical school, how many research projects have you worked on?**

- ○ 1
- ○ 2
- ○ 3
- ○ 4
- ○ 5
- ○ Other (please specify in the box below)

____

Q20 **Since starting medical school, I have gained research opportunities through the following**
(Please tick all that apply)

- ☐ Summer research electives
- ☐ Mandatory curricular study modules
- ☐ Optional curricular study modules
- ☐ Extracurricular term-time research activities
- ☐ Intercalation
- ☐ Other (please specify in the box below)

____

Q21 **Since starting medical school, as part of a research project, I have been involved in**
(Please tick all that apply)

- ☐ Formulating a research question
- ☐ Conducting a structured literature search
- ☐ Designing methodology
- ☐ Applying for ethical approval
- ☐ Data collection (quantitative and/or qualitative data)
- ☐ Data analysis (quantitative and/or qualitative data)
- ☐ Presenting results (oral or poster presentations)
- ☐ Writing reports/ manuscripts
- ☐ Other (please specify in the box below)

____

End of Block: Research engagement

Start of Block: Research self-efficacy

Q22 **Please rate how confident you are to execute the following research-related actions**

	Not at all	A little bit	Average	Fairly	Very
Formulating a research question	○	○	○	○	○
Conducting a structured literature search	○	○	○	○	○
Designing methodology	○	○	○	○	○
Applying for ethical approval	○	○	○	○	○
Data collection (quantitative and/or qualitative data)	○	○	○	○	○
Data analysis (quantitative and/or qualitative data)	○	○	○	○	○
Presenting results (oral or poster)	○	○	○	○	○
Writing reports/ manuscripts	○	○	○	○	○

End of Block: Research self-efficacy

Start of Block: Perceptions of research

Q23 **Please select the extent to which you agree with each statement**

	Strongly disagree	Disagree	Neither agree nor disagree	Agree	Strongly agree
It is important for medical professionals to have scientific skills	○	○	○	○	○
A doctor should be able to independently complete/ take part in research	○	○	○	○	○
Training in scientific research should be a mandatory component of undergraduate medical education	○	○	○	○	○
A scientific educational programme is important for me	○	○	○	○	○
I enjoy the research components of my course	○	○	○	○	○

End of Block: Perceptions of research

Start of Block: Career Interest

Q24 **Please rate your level of interest in being involved in research after graduating from medical school**

◯ Strongly interested

◯ Interested

◯ Neither interested nor uninterested

◯ Uninterested

◯ Strongly uninterested

Q25
Are you interested in clinical academic posts such as the **Academic Foundation Programme (AFP), Academic Clinical Fellowship (ACF) or Clinical Lectureship (CL)?**

◯ Yes

◯ No

◯ Undecided

End of Block: Career Interest

Start of Block: Publications

Q26 **Have you been listed as an author in any PubMed-indexed article that resulted from work(s) that you contributed to since starting medical school? (excluding collaborator-status)**

◯ Yes

◯ No

Display This Question:

If Have you been listed as an author in any PubMed-indexed article that resulted from work(s) that y... = Yes

Q27 **What type of publication(s) are they?**
(Please tick all that apply)

- ☐ Original article
- ☐ Protocols
- ☐ Systematic review and/or meta-analysis
- ☐ Narrative review/ literature review
- ☐ Case report
- ☐ Letter
- ☐ Other (please specify in the box below)

____

Display This Question:

If Have you been listed as an author in any PubMed-indexed article that resulted from work(s) that y... = Yes

Q28 **From work(s) done since starting medical school, on how many PubMed-indexed publications are you listed as an author (excluding collaborator status)?**

- ○ 1
- ○ 2
- ○ 3
- ○ 4
- ○ 5
- ○ Other (please specify in the box below)

____

Display This Question:

If Have you been listed as an author in any PubMed-indexed article that resulted from work(s) that y... = Yes

Q29 **From work(s) done since starting medical school, on how many PubMed-indexed articles are you listed as the first (or joint-first) author?**

- ◯ 0
- ◯ 1
- ◯ 2
- ◯ 3
- ◯ 4
- ◯ 5
- ◯ Other (please specify in the box below)

____

Q30 **Have you been listed as a collaborator in any PubMed-indexed article that resulted from work(s) that you contributed to since starting medical school?**

- ◯ Yes
- ◯ No

Display This Question:

If Have you been listed as a collaborator in any PubMed-indexed article that resulted from work(s) t... = Yes

Q31 **From work(s) done since starting medical school, on how many PubMed-indexed articles are you listed as a collaborator?**

○ 1

○ 2

○ 3

○ 4

○ 5

○ Other (please specify in the box below)

____

End of Block: Publications

Start of Block: Presentations

Q32 **From work(s) done since starting medical school, have you presented a poster/oral presentation at a conference? This could be at local/regional, national, or international levels.**

○ Yes

○ No

Display This Question:

If From work(s) done since starting medical school, have you presented a poster/oral presentation at... = Yes

Q33 **How many poster presentations? NB if you presented the same abstract as a poster at two or more different conferences, this counts as one poster presentation**

○ 0

○ 1

○ 2

○ 3

○ 4

○ 5

○ Other (please specify in the box below)

____

Display This Question:

If From work(s) done since starting medical school, have you presented a poster/oral presentation at... = Yes

Q34 **How many oral presentations? NB if you presented the same abstract orally at two or more different conferences, this counts as one oral presentation.**

○ 0

○ 1

○ 2

○ 3

○ 4

○ 5

○ Other (please specify in the box below)

____

Display This Question:

If From work(s) done since starting medical school, have you presented a poster/oral presentation at... = Yes

Q35 **Please select the highest level at which you have presented (oral and/or poster presentation)**

- ○ International meeting/conference
- ○ National meeting/conference
- ○ Regional/local meeting/conference
- ○ Not sure

End of Block: Presentations

Start of Block: Research-related grants/ bursaries

Q36 **Since starting medical school, have you applied for any grant/bursary to undertake a research-related activity (such as research projects, attending conferences, presentations, publications, intercalated degree etc)? (Excluding NHS bursary and Student Finance)**

- ○ Yes
- ○ No
- ○ Prefer not to answer

Display This Question:

If Since starting medical school, have you applied for any grant/bursary to undertake a research-rel... = Yes

Q37 **How many research-related grant/bursary applications have you submitted and have been told the outcome?**

- ◯ 1
- ◯ 2
- ◯ 3
- ◯ 4
- ◯ 5
- ◯ Other (please specify in the box below)
____
- ◯ Prefer not to say

Display This Question:

If Since starting medical school, have you applied for any grant/bursary to undertake a research-rel... = Yes

Q38 **How many of these grant/bursary applications were successful?**

- ◯ 0
- ◯ 1
- ◯ 2
- ◯ 3
- ◯ 4
- ◯ 5
- ◯ Other (please specify in the box below)
____
- ◯ Prefer not to say

End of Block: Research-related grants/ bursaries

Start of Block: Motivation

*

Q39 **In terms of research opportunities, I would like to be involved in** (Please tick all that apply)

- ☐ Formulating a research question
- ☐ Conducting a structured literature search
- ☐ Designing methodology
- ☐ Applying for ethical approval
- ☐ Data collection (quantitative and/or qualitative data)
- ☐ Data analysis (quantitative and/or qualitative data)
- ☐ Presenting results (oral and/or poster)
- ☐ Writing reports/ manuscripts
- ☐ Other (please specify) ____
- ☐ None

*

Q40

What motivates you to engage in research?

(Please tick all that apply)

- ☐ Intellectually stimulating
- ☐ Interest in scientific enquiry
- ☐ To contribute to improving patient care
- ☐ To learn new skills
- ☐ To facilitate my career progression (eg job applications)
- ☐ Advice from tutor/ mentor
- ☐ Peer pressure
- ☐ Previous productive research (e.g. presentation, publication, prize etc)
- ☐ Other (please specify in the box below)

____

- ☐ No interest in research

End of Block: Motivation

Start of Block: Barriers

Q41 **What do you think is/are your main barrier(s) to engaging in research?**
(Please tick all that apply)

- ▢ Time constraint
- ▢ Difficulty finding research supervisors
- ▢ Limited awareness of opportunities
- ▢ My lack of interest in research
- ▢ Poor support and/or guidance from supervisor
- ▢ Inadequate training in research methodology
- ▢ Lack of productivity (e.g. publication, presentation, prize) in my previous research projects
- ▢ Lack of acknowledgement for my work on previous projects
- ▢ Lack of funding
- ▢ Perceived lower salary of academic clinicians
- ▢ Other (please specify in the box below) ____
- ▢ None

End of Block: Barriers

Start of Block: Suggested modes of support for research engagement

Q42 What forms of support would encourage you to get involved in research and/or reduce your **barriers** to engage in research?
(Please tick all that apply)

- ☐ Financial support to undertake research-related activities
- ☐ Formal recognition (e.g. prizes, dean's letter) of student's engagement in research
- ☐ Formal teaching and/or e-learning resources to equip you with skills and knowledge to engage in research
- ☐ A local research officer/society/division that provides regular updates on research opportunities within the medical school and other institutions, sources of funding, conferences, prizes and awards
- ☐ More guidance on how to become an academic clinician and how to get involved in research in medical school and subsequently
- ☐ A directory that provides guidance on how to approach and connect with research mentors and/or supervisors within the medical school and other institutions
- ☐ Increased availability of research projects aimed specifically at students with little/no previous research experience
- ☐ More opportunities to publish your work (e.g. in medical student journals)
- ☐ Other (Please specify in the box below)
____
- ☐ None

End of Block: Suggested modes of support for research engagement

---

APPENDIX: List of Russell Group medical schools

- University of Birmingham
- University of Bristol
- University of Cambridge
- Cardiff University
- University of Edinburgh

- University of Exeter
- University of Glasgow
- Imperial College London
- King's College London
- University of Leeds
- University of Liverpool
- University of Manchester
- Newcastle University
- University of Nottingham
- University of Oxford
- Queen Mary, University of London
- Queen's University Belfast
- University of Sheffield
- University of Southampton
- University College London
- University of Warwick
